# A Novel Turn-On Fluorescence Probe Based on Cu(II) Functionalized Metal–Organic Frameworks for Visual Detection of Uric Acid

**DOI:** 10.3390/molecules27154803

**Published:** 2022-07-27

**Authors:** Jie Yang, Jie Che, Xin Jiang, Yangchun Fan, Daojiang Gao, Jian Bi, Zhanglei Ning

**Affiliations:** College of Chemistry and Materials Science, Sichuan Normal University, Chengdu 610068, China; yangjie_deyouxiang@163.com (J.Y.); chejie2022@163.com (J.C.); jiangxin20220706@163.com (X.J.); fanyc0702@163.com (Y.F.); daojianggao@sicnu.edu.cn (D.G.); bijian686@126.com (J.B.)

**Keywords:** metal–organic frameworks, probe, detection, uric acid, logic gate

## Abstract

As an important biomarker in urine, the level of uric acid is of importance for human health. In this work, a Cu(II) functionalized metal–organic framework (Cu^2+^@Tb-MOFs) is designed and developed as a novel fluorescence probe for wide-range uric acid detection in human urine. The study shows that this fluorescence platform demonstrated excellent pH-independent stability, high water tolerance, and good thermal stability. Based on the strong interaction between metal ions and uric acid, the designed Cu^2+^@Tb-MOFs can be employed as efficient turn-on fluorescent probes for the detection of uric acid with wide detection range (0~10^4^ µM) and high sensitivity (LOD = 0.65 µM). This probe also demonstrates an anti-interference property, as other species coexisted, and the possibility for recycling. The sensing mechanisms are further discussed at length. More importantly, we experimentally constructed a molecular logic gate operation based on this fluorescence probe for intelligent detection of uric acid. These results suggest the Cu(II) functionalized metal–organic framework can act as a prominent candidate for personalized monitoring of the concentration of uric acid in the human urine system.

## 1. Introduction

Uric acid (2,6,8-trihydroxypurine, UA), a major metabolite in birds and mammals, is the ultimate metabolized product of purine nucleotide metabolism in body fluid [1,2]. An abnormal level of UA will affect the physiological system and even other normal functions. A risen level of UA causes gout, chronic kidney disease, hyperuricemia, hypertension, cardiovascular disease, etc. [3,4]. The content of UA in serum and urine has become an important indicator for the prediction and diagnosis of diseases. Therefore, the sensitive and precise determination of UA is significant in, for instance, disease screening and physiological studies [5]. In recent years, many methods have been developed for the estimation and detection of UA levels, such as enzymatic assays [6,7], electrochemical sensors [8,9,10,11], spectrophotometric methods [12,13,14,15], Raman spectroscopy [16], chromatography [17,18,19], and fluorescent spectrometry [20,21,22,23,24]. Although enzymatic assays are the conventional method for the detection of UA, this method has challenges, including the high purification costs and the thermal instability of enzyme. In addition, most of these methods generally have some intrinsic disadvantages, such as the use of expensive instruments and time-consuming and laborious manipulation procedures, which greatly limit their practical and wide applications in the determination of UA. Thus, it is crucial to develop a simple, precise and low-cost method for identification of UA.

Fluorescent probes have been rapidly developed and have widely received increasing attention [25,26,27,28], because of their distinctive advantages, including easy modification, adjustable luminescence, good biocompatibility, facile operation, use of simple instrumentation, excellent analysis sensitivity, fast response, and highly sensitive fluorescence. Owing to their prominent fluorescence properties, lanthanide rare-earth metal–organic frameworks have been made for UA detection [29,30,31]. However, most of these cannot detect UA over other common components in human urine. In addition, few of these have a noteworthy fluorescence enhancement response to UA. Therefore, the fabrication of a fluorescent probe for UA with outstanding selectivity and high sensitivity is of great challenge.

Logic gates are the basic components of integrated circuits for information processing and storage [32,33]. If molecules are used to describe the input and output signals in a logic gate and thus realize the logic operations at the molecular level, such a logic system is called a molecular logic gate [34,35]. In recent years, the field has evolved from a single physical or chemical input to a combinatorial and sequential operation, showing great potential and broad promise. Molecular logic gates are gradually replacing traditional semiconductor electronic computers with their significant advantages and are used for heavy metal ion detection, environmental monitoring, food safety detection, pre-disease diagnosis, and biosensor research [36,37,38]. MOFs-based fluorescent probes are highly selective and sensitive, allowing the construction of chemical sensors for sensing various analytes by host–guest interactions. Due to the different fluorescence changes (such as “quenching” or “enhancement” effect) of MOFs [39] in detecting analytes, molecular logic gates can be further constructed for programmed detection.

**Scheme 1:**
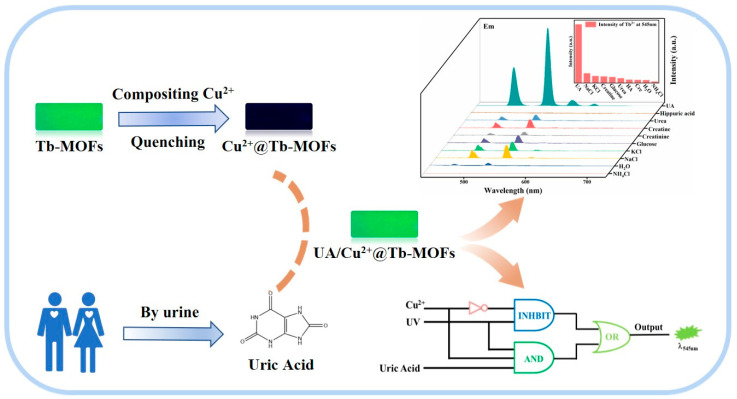
Schematic diagram of UA detection and application.

Here, in this work, we synthesize a novel fluorescence nanoprobe based on Cu(II) functionalized metal–organic frameworks (Cu^2+^@Tb-MOFs) and explore its application in fluorescence detection (Scheme 1). Ten kinds of related substance in urine were detected, and their impacts on the fluorescence of the Cu^2+^@Tb-MOFs compound was analyzed. The Cu^2+^@Tb-MOFs can specially and selectively recognize UA by fluorescence recovering and exhibit high sensitivity for UA. In addition, a molecular logic gate was constructed based on the whole system, and finally a molecular logic network system for uric acid detection connecting basic and integrated logic operations was implemented. This strategy is simple and practical, and provides a guiding method for constructing molecular-level logic gates for uric acid detection on a simple platform.

## 2. Results and Discussion

### 2.1. Characterization of Cu^2+^@Tb-MOFs Fluorescent Probe

**Figure 1:**
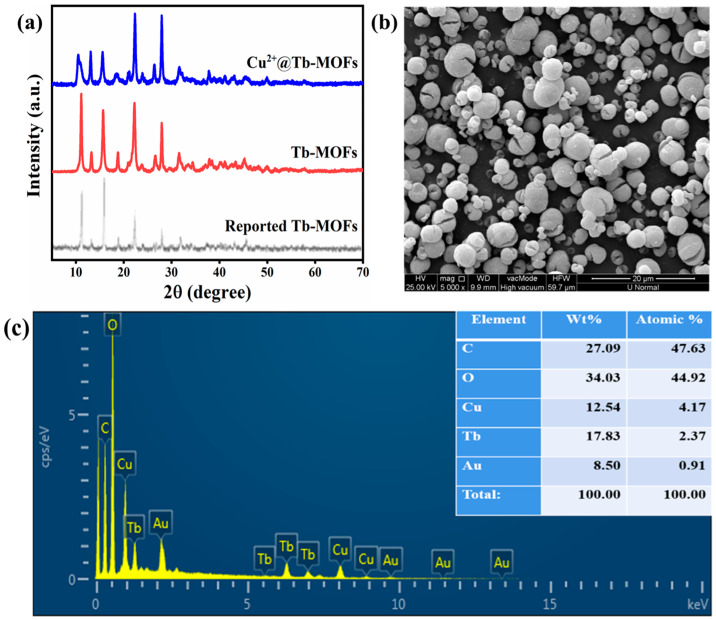
(**a**) PXRD patterns of the reported Tb-MOFs, the synthetic crystalline Tb-MOFs and Cu^2+^@Tb-MOFs samples; (**b**) SEM image and (**c**) EDX of Cu^2+^@Tb-MOFs samples.

The powder X-ray diffraction patterns (PXRD) of the reported Tb-MOFs, the synthetic crystalline Tb-MOFs and Cu^2+^@Tb-MOFs samples are shown in Figure 1a. The main diffraction peaks of the prepared Tb-MOFs match well with that of the reported one [40], suggesting that the pure Tb-MOFs samples can be synthesized by this fast and facile method at room temperature. Moreover, the structure of the as-obtained Cu^2+^@Tb-MOFs is also verified by XRD. Compared with the Tb-MOFs, the positions of the diffraction peaks of Cu^2+^@Tb-MOFs are basically the same and the intensity varies slightly, indicating that the addition of Cu^2+^ would not cause a structural change in the Tb-MOFs samples. Moreover, the Cu^2+^@Tb-MOFs samples were immersed in water, for 12 h and 24 h, or in an environment with different pH values (pH = 3.0~9.0) to test their corresponding XRD patterns. As shown in Figure S1, the crystal structure has hardly changed, indicating that the composite Cu^2+^@Tb-MOFs have good structural stability in different pH and water environments. The thermogravimetric analysis of Cu^2+^@Tb-MOFs and Tb-MOFs (Figure S2a) show that there are about three weight-loss intervals. The weight loss of (14.37%) Tb-MOFs sample at the first stage appears at a platform before 200 °C, which is mainly due to the loss of the free water and coordination water molecules in the system. In the second stage, at 200~400 °C, the weight loss (30.9%) is due to the lack of ligand mucus acid. At the third stage, the final weight loss (25.85%) after 400 °C may be due to the pyrolysis of the whole system. The result basically corresponded to the theoretical weight loss rates of the assumed structure of Tb-MOFs. Moreover, the weight loss rates of Cu^2+^@Tb-MOFs in three stages are 15.43%, 26.73% and 24.93%, respectively. It can be also seen that the structure of Cu^2+^@Tb-MOFs and Tb-MOFs are the similar. In addition, from the thermal decomposition rate by DTG curve (Figure S2b), it can be observed that the unmodified material Tb-MOFs (orange) and the copper ion modified material Cu^2+^@Tb-MOFs (green) have similar pyrolysis at about 200 °C. As the pyrolysis temperature is about 400 °C, Tb-MOFs has obvious pyrolysis, whereas Cu^2+^@Tb-MOFs does not have this process, indicating that Cu^2+^@Tb-MOFs has better high-temperature anti-pyrolysis ability.

Morphology of Cu^2+^@Tb-MOFs was studied by scanning electron micrograph (SEM). As shown in Figure 1b, the Cu^2+^@Tb-MOFs samples are composed of a large number of cracked spheres with a diameter of 3–5 μm. Comparing the SEM image of Tb-MOFs samples (Figure S3a), the morphology of Cu^2+^@Tb-MOFs samples had not changed significantly, which is consistent with XRD results. Subsequently, the composition changes of Cu^2+^@Tb-MOFs (Figure 1c) and Tb-MOFs (Figure S3b) were measured by energy-dispersive X-ray analysis (EDX). The Cu^2+^@Tb-MOFs samples contained several elements of Tb, C, H, O, and Cu (except Au) and the Tb-MOFs sample contained several elements of Tb, C, H, O (except Au). It can be seen that Cu element is detected in the Cu^2+^@Tb-MOFs samples, and the mole ratio of Cu and Tb (Cu:Tb = 1.8) is close to the stoichiometric ratio of the addition amount (Cu:Tb = 2.0). Moreover, the loading levels of Cu^2+^ onto the Tb-MOFs samples have been further evaluated by inductively coupled plasma (ICP) analysis. The experimental molar ratio of Cu/Tb ions in Cu^2+^@Tb-MOFs was checked to be 1.81, which is also consistent with the analysis result of Cu^2+^@Tb-MOFs.

The emission spectra of Tb-MOFs and Cu^2+^@Tb-MOFs are presented in Figure S4. Under the excitation of 227 nm at room temperature, several emission spectra (EM) of Tb^3+^ appeared at 450~700 nm, belonging to ^5^D_4_→^7^F_j_ (j = 6, 5, 4, 3) [41,42], respectively. The maximum emission wavelength is 545 nm (^5^D_4_→^7^F_5_), which in principle leads to green emission [43]. However, the Cu^2+^@Tb-MOFs overall fluorescence emission peak is considerably lower than the normal level of the original Tb-MOFs, so the actual green fluorescence does not appear under UV irradiation. In addition, the fluorescence stability of Cu^2+^@Tb-MOFs in water and different pH environments was also studied. There was no significant change in the fluorescence spectrum and the corresponding fluorescence intensity histogram (Figure S5), indicating that Cu^2+^ was locked in the composite Cu^2+^@Tb-MOFs and had high stability in different pH and water environments. The results show that Cu^2+^@Tb-MOFs has good pH and water stability and can adapt to various environments, which makes the fluorescent sensor better in practical application.

### 2.2. Detection of Uric Acid in Aqueous Solutions

**Figure 2:**
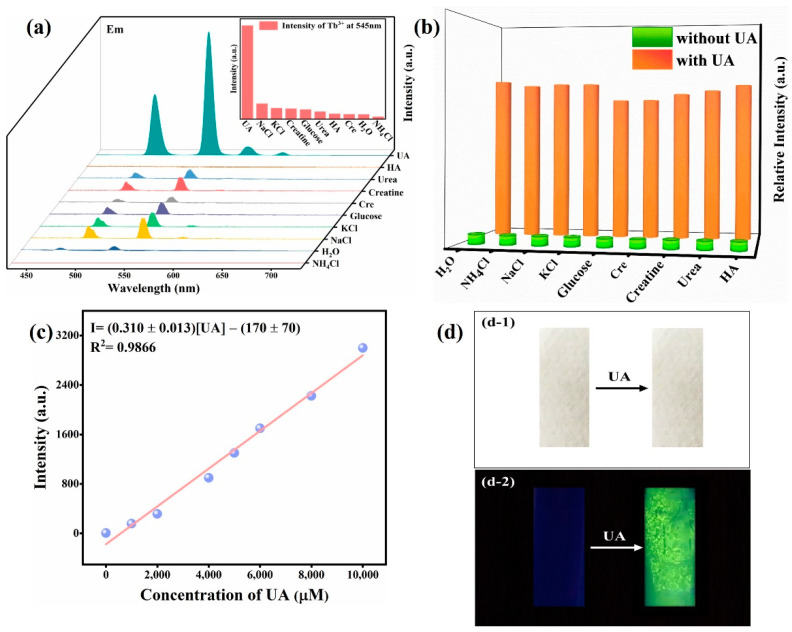
(**a**)The emission spectra of Cu^2+^@Tb-MOFs sample materials immersed in various urine composition solutions (the inset is 2D-histogram based on the intensity of ^5^D_4_→^7^F_5_ transition of Tb^3+^ in Cu^2+^@Tb-MOFs toward various urine chemicals, λex = 337 nm); (**b**) The fluorescence intensity of Cu^2+^@Tb-MOFs in the presence of other components in urine (green bars), and the subsequent addition of UA (orange bars); (**c**) The linear relationship the fluorescence intensity and the concentration of UA; (**d**) Comparison of Cu^2+^@Tb-MOFs test paper for uric acid recognition under light (d-1) and UV (d-2) irradiation.

To test the potential of Cu^2+^@Tb-MOFs as fluorescent probes for uric acid, it was immersed in various aqueous solutions of common components in human urine, including uric acid (UA), NaCl, KCl, creatine, glucose (Glu), urea, hippuric acid (HA), creatinine (Cre), NH4Cl, and H2O. Figure 2a shows the fluorescence emission spectra of Cu^2+^@Tb-MOFs sample materials immersed in aqueous solutions of different urine components after sonicating for 20 min. The results show that only uric acid induced a remarkable rebound of the fluorescence spectrum of Cu^2+^@Tb-MOFs showing a turn-on response, whereas other urine chemicals showed almost no changes. The inset shows the fluorescence measurement of Cu^2+^@Tb-MOFs in suspension state, the fluorescence intensity 2D-histogram showed that after the addition of UA solution to the composite Cu^2+^@Tb-MOFs, the fluorescence intensity of Tb^3+^ at 545 nm was restored and was considerably higher than that of other urine components, suggesting the composite material Cu^2+^@Tb-MOFs has a good selectivity for UA in biological metabolites. Therefore, the composite Cu^2+^@Tb-MOFs has specific recognition for the detection of UA in aqueous solution and high selectivity for UA.

The anti-interference of fluorescent nanoprobes is a very significant characteristic of their practical analytical performance. Actual biological samples (in human urine) contain a large number of other molecules that coexist and compete with each other. Therefore, in order to test the effect of the coexistence of other competitive substances on the identification capability of Cu^2+^@Tb-MOFs, the competitive uric acid response of Cu^2+^@Tb-MOFs probe in the presence of potentially interfering species in urine was studied under the same experimental conditions. As shown in Figure 2b, as other biological metabolites (except UA) were added, the fluorescence of the composites did not recover, and the intensity did not change significantly; whereas, the luminescence of the composite at 545 nm was restored by the addition of UA, and its fluorescence intensity was significantly improved compared with that before the addition of uric acid. This indicates that other common biological metabolites have little influence on the luminescence of the composite, and that the composite has superior selectivity and strong anti-interference ability for UA detection.

The sensitivity of fluorescent nanoprobes is also one of the important factors in measuring the comprehensive properties of composite materials. Concentration-dependent luminescence was carried out in aqueous solution. The fluorescence response test of the composite material Cu^2+^@Tb-MOFs was explored for the quantitative detection of uric acid in aqueous solution. The fluorescence titration spectra are showed in Figure S6. It can be clearly that the emission intensity of Cu^2+^@Tb-MOFs at 545 nm gradually increased with incremental UA concentration. As shown in Figure 2c, the emission intensity of composite material Cu^2+^@Tb-MOFs has a good linear relationship with UA concentration in the range of 0~10^4^ µM (correlation coefficient R^2^ = 0.9866). Linear fitting equation: *I* = (0.310 ± 0.013) *[UA]* − 174.2 (170 ± 70) (*[UA]* is the uric acid concentration in the equation, and *I* is the fluorescence intensity of uric acid aqueous solutions with different concentrations). According to the IUPAC 3*σ* criterion formula: 3*σ*/*K* (*σ* is the standard deviation of 20 repeated fluorescence measurements of blank solution, *K* is the slope of the fitting line). The detection limit (LOD) [44] is calculated to be 0.65 µM, which is much lower than the normal concentration of uric acid in blood (0.21~0.42 mM) and urine (0.95~4.50 mM) [16]. This linear relationship can be used to quantitatively measure unknown concentrations of UA in real biological samples. So, the composite MOFs Cu^2+^@Tb-MOFs is a fluorescence monitoring material with excellent response to UA, and that has excellent selectivity and high sensitivity. In addition, the performance comparison of different nanoprobes for uric acid detection is listed in Table 1. Comparing different probes, Cu^2+^@Tb-MOFs has a wider detection range and a lower LOD value, which makes its performance comparable or better than other probes reported in the literature.

In order to make the fluorescence sensor easier and more intuitive in practical application, it is designed as a portable test paper for uric acid detection. As shown in Figure 2d, the test paper was immersed in uric acid solutions, and the results could be directly observed under UV irradiation (Figure 2d, d-2) after drying in the air. The test paper showed a dull color with almost no fluorescence before being soaked in uric acid solution, whereas the test paper treated with uric acid solution showed a bright green color. Thus, the fluorescence color of the test paper can be directly distinguished by the naked eye. General information about uric acid concentration can be obtained, which can be used for preliminary detection to determine whether the related diseases are caused by high uric acid. Therefore, the composite probe based on Cu^2+^@Tb-MOFs showed excellent performance in the detection of uric acid.

Recyclable performance plays an important role in practical applications when composite materials are used as sensors. To investigate the reversibility of Cu^2+^@Tb-MOFs material, the fluorescence intensity of Cu^2+^@Tb-MOFs at 545 nm was monitored. After completing the test of UA detection, Cu^2+^ was added into the composite solution for the next measurement. As illustrated in Figure S7, the fluorescence intensity of Cu^2+^@Tb-MOFs recovered as a result of UA addition, whereas the intensity decreased when Cu^2+^ existed. After four recycles, performed by successive addition of UA and Cu^2+^, the fluorescence intensity of the Cu^2+^@Tb-MOFs material sensor obtained changed little compared with that of the initial cycle, which indicates that the Cu^2+^@Tb-MOFs material sensor can be used for recyclable detection of UA.

### 2.3. Sensing Mechanism

**Figure 3:**
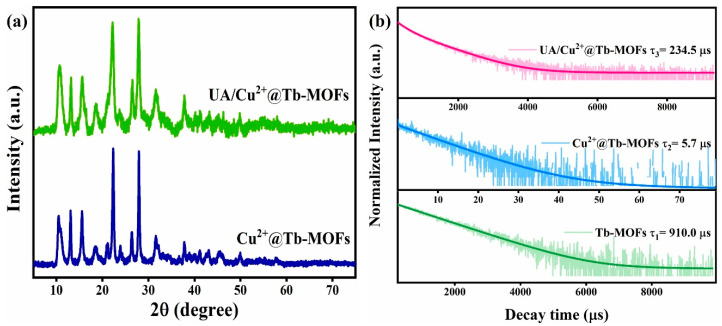
(**a**) PXRD pattern of Cu^2+^@Tb-MOFs and UA/Cu^2+^@Tb-MOFs; (**b**) Luminescence decay curve of Tb-MOFs, Cu^2+^@Tb-MOFs and UA/Cu^2+^@Tb-MOFs at 545 nm.

The possible mechanism of the Cu^2+^@Tb-MOFs sample for UA detection was investigated in detail. As shown in Figure 3a, the powder X-ray diffraction pattern of UA/Cu^2+^@Tb-MOFs collected from UA aqueous solution is in good agreement with that of Cu^2+^@Tb-MOFs and Tb-MOFs. This result not only indicates the structural stability of Cu^2+^@Tb-MOFs, but also excludes the possibility of fluorescence recovery caused by structural collapse or reorganization [48,49]. The luminescence lifetime is a very important parameter to explore the fluorescence recovery mechanism of Cu^2+^@Tb-MOFs; we determined the fluorescence lifetime of Tb-MOFs, Cu^2+^@Tb-MOFs and UA/Cu^2+^@Tb-MOFs samples. As shown in the Figure 3b, the fluorescence lifetime of the generated Cu^2+^@Tb-MOFs (5.7 μs) decreases greatly compared with that of Tb-MOFs (910.0 μs). After adding UA to Cu^2+^@Tb-MOFs, the fluorescence life of UA/Cu^2+^@Tb-MOFs was partially recovered and enhanced (from 5.7 μs to 234.5 μs) [50]. This restored emission lifetime indicates the interaction between UA sites and Cu^2+^ promotes the recovery of the fluorescent probes [51].

**Figure 4:**
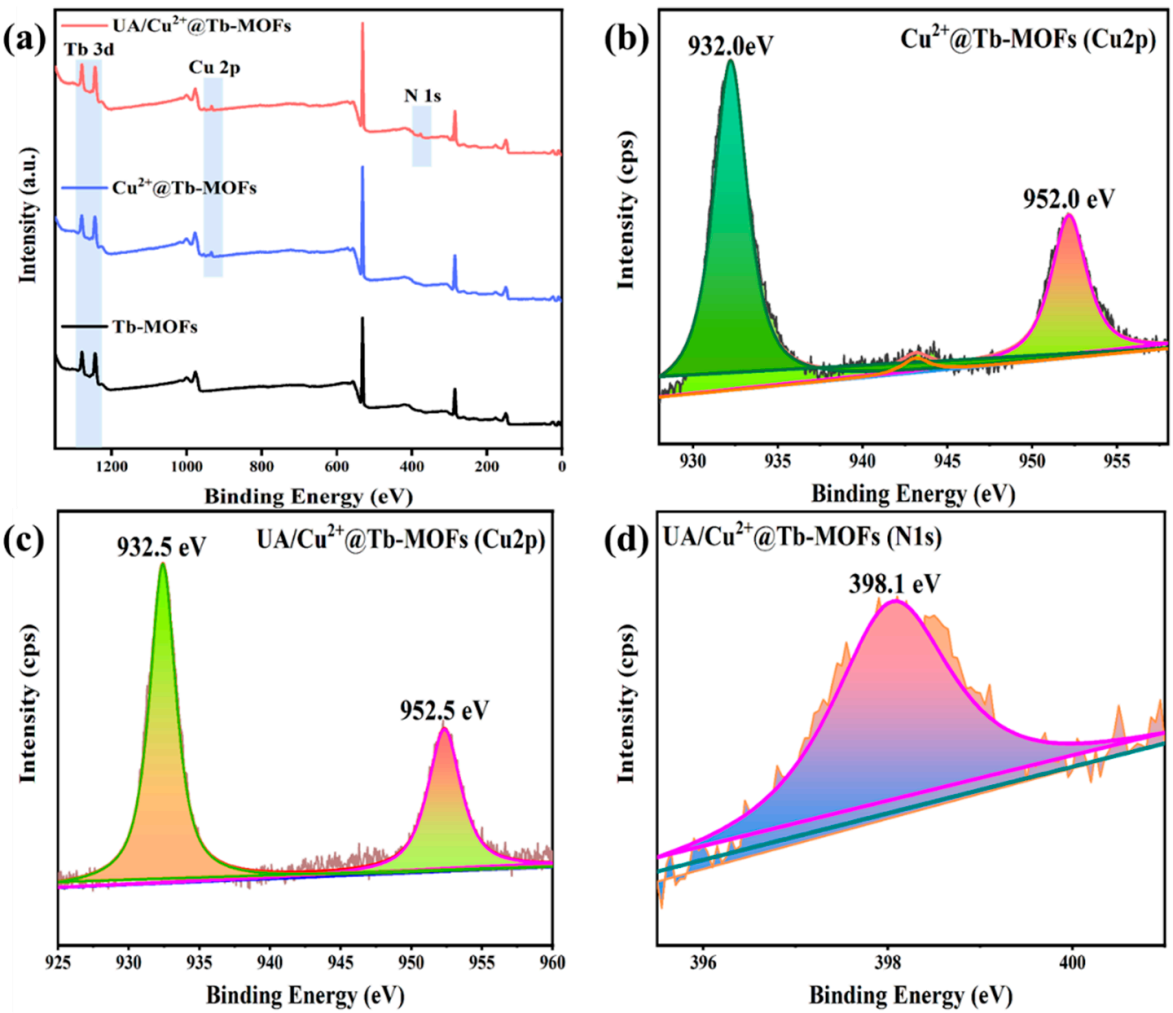
(**a**) The XPS spectra for Tb-MOFs, Cu^2+^@Tb-MOFs, UA/Cu^2+^@Tb-MOFs; (**b**) The XPS peaks of Cu^2+^ in Cu^2+^@Tb-MOFs samples; (**c**) The XPS peaks of Cu^2+^ in UA/Cu^2+^@Tb-MOFs samples; (**d**) The XPS peaks of N in UA/Cu^2+^@Tb-MOFs samples.

The XPS patterns of Tb-MOFs, Cu^2+^@Tb-MOFs and UA/Cu^2+^@Tb-MOFs are shown in Figure 4. After the introduction of UA, the peak position of Cu 2p of UA/Cu^2+^@Tb-MOFs exhibits a higher binding energy (932.5 and 952.5 eV) compared to that of Cu^2+^@Tb-MOFs (932.0 and 952.0 eV) (Figure 4b,c), which is also direct proof of the interaction between Cu^2+^ and UA in Cu^2+^@Tb-MOFs [34]; whereas, a new peak position of N 1s (Figure 4d) appeared in the XPS sub-peak of UA/Cu^2+^@Tb-MOFs. Considering N element does not exist in Cu^2+^@Tb-MOFs structure, the signal of N 1s of UA/Cu^2+^@Tb-MOFs could be caused by the addition of uric acid (Figure S8), giving another powerful proof of successful coordination. Therefore, it can be speculated that, after the addition of uric acid recognition, Cu^2+^ reacts with uric acid in the UA/Cu^2+^@Tb-MOFs system fixed on the surface or in the holes of MOFs.

The mechanism of fluorescence recovery was further studied. Shown in Figure S9 are the fluorescence spectra of Tb-MOFs in water (a) and uric acid (d), as well as the fluorescence spectra of Cu^2+^@Tb-MOFs suspension with uric acid (b) and without uric acid (c). The fluorescence spectrum of UA/Tb-MOFs formed by Tb-MOFs combining with uric acid was similar to that of the initial Tb-MOFs. Therefore, uric acid did not affect the fluorescence of Tb-MOFs. However, Cu^2+^@Tb-MOFs composites have almost no fluorescence emission, and Cu^2+^ has an obvious quenching effect on the 4f–4f transition of Tb^3+^. The fluorescence intensity recovered when uric acid was added to Cu^2+^@Tb-MOFs suspension. As shown in Figure S10, under UV irradiation, the observed results of the corresponding substance are consistent with the change of fluorescence intensity in Figure S9. Compared with the Cu^2+^@Tb-MOFs, the fluorescence intensity of UA/Cu^2+^@Tb-MOFs formed by UA doping was significantly recovered, and under UV lamp irradiation, the results can be clearly identified with the eye.

In order to further explore the mechanism of fluorescence recovery of Cu^2+^@Tb-MOFs composite to UA, the visible absorption spectra of a Cu^2+^ solution and a mixed solution of Cu^2+^ and UA were tested. As shown in Figure S11, compared with the visible absorption spectra of the Cu^2+^ solution, the absorption peaks of the mixed solution of Cu^2+^ and UA have changed, further indicating that there is an intense reaction between Cu^2+^ and UA [52]. Therefore, based on the above experiments, it can be reasonably inferred that the interaction between Cu^2+^ and UA is the main factor. The nitrogen/oxygen-containing group in the structure of uric acid was employed as a strong Cu^2+^ chelator to Cu^2+^@Tb-MOFs composites, and Cu^2+^ was removed from the surface of Cu^2+^@Tb-MOFs composites, leading to fluorescence recovery of Cu^2+^@Tb-MOFs composites.

### 2.4. Construction of Luminescent Logic Gate

**Figure 5:**
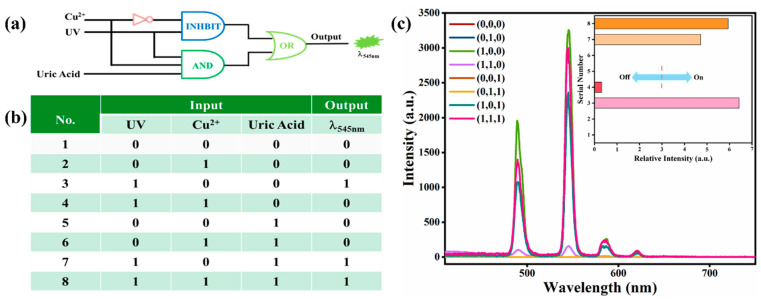
(**a**) Electronic equivalent circuitry of the logic gate; (**b**) The truth table of the logic gate; (**c**) Emission spectra of the logic gates (the inset is the relative emission intensity at 545 nm).

Based on the successful observation of Cu^2+^@Tb-MOFs selective analysis ability toward UA, we have developed a fluorescent logic gate system capable of multipath analysis of UA substance in the system. In the logical operation, Tb-MOFs act as gates, while the substance to be detected (Cu^2+^, UA and the necessary premise of UV) and fluorescence emission changes serve as chemical input and output at 545 nm (λ_545nm_), respectively (Figure 5). As shown in Figure 5a, the input is “off” (i.e., 0) when the detection substances (Cu^2+^, UA and UV) are not injected and the input is “on” (i.e., 1) when the substances are added. The output value is defined as 1 (fluorescence recovery “ON”) or 0 (fluorescence quenching “OFF”) by comparing with the output threshold (fluorescence emission relative intensity at 545 nm). Then, INHIBIT, AND, and OR logical operations are driven by different input scenarios. The truth table (Figure 5b) has 8 input cases, among which the output is 1 when the input is (1/0/0, 1/0/1, 1/1/1) and 0 when the input is other cases. It can be seen from Figure 5c that the threshold of the system is related to the fluorescence intensity at 545 nm emission center. The threshold is expressed as the relative intensity below and above 3 under different input conditions. The output is “1” when the relative intensity is higher than the threshold value, whereas the output is “0” when the relative intensity is lower than the threshold value, and the output response signals can be observed by the naked eye as fluorescence recovery and fluorescence quenching, respectively. This molecular logic gate sensor can directly carry out visual detection of UA through logic operation and analyze the changes of these different inputs. To the best of our knowledge, this is the first example of the design of a logic gate system for detecting uric acid using Cu(II) functionalized metal–organic frameworks.

## 3. Experimental Section

### 3.1. Reagents and Instruments

All chemical reagents and solvents are commercially available.

### 3.2. Synthesis of Tb-MOFs and Cu^2+^@Tb-MOFs

The Tb-MOFs (Tb(L)_1.5_·5H_2_O, L = C_6_H_10_O_8_) sample was synthesized according to our previous report via a modified procedure [53]. Firstly, 0.4 mmol mucic acid was dissolved in 20 mL of distilled water and vigorously stirred for 20 min. Then, 2.5 mL of 0.35 M KOH solution was added into the solution and stirred vigorously for 30 min. After the solution was completely dissolved, a transparent solution was obtained. Then, 8 mL of 0.025 M Tb(NO_3_)_3_ solution was added. The whole reaction process was kept at room temperature with vigorous stirring until the reaction stopped after 30 min. The resulting precipitates were washed alternately with distilled water and ethanol solution several times, and then dried for 24 h in an oven at 55 °C.

The Cu^2+^@Tb-MOFs sample was synthesized using a simple ultrasonic immersion method. Firstly, the as-synthesized Tb-MOFs were immersed in 1 mM aqueous solution of Cu^2+^ and then the mixture was shaken uniformly and equilibrated evenly for 30 min by ultrasound treatment. Finally, the precipitates obtained were collected centrifugally and dried in an oven for 24 h.

### 3.3. Fluorescence Sensing of Detection UA

In a typical process for sensing urine chemicals, 3.0 mg of Cu^2+^@Tb-MOFs powders were simply added into the aqueous solutions (5 mL, 10 mM) of different urine chemicals, including uric acid (UA), NaCl, KCl, creatine, glucose (Glu), urea, hippuric acid (HA), creatinine (Cre), NH_4_Cl, and H_2_O. The luminescence spectra of these suspensions were measured after sonicating for 20 min. Each measurement was repeated three times, and the average value was used. In the selectivity experiment, 2 mL of the Cu^2+^@Tb-MOFs suspension was added into a mixture containing both interfering analyte and UA.

The pH value and water stability of Cu^2+^@Tb-MOFs were determined by soaking 20 mg Cu^2+^@Tb-MOFs powder in 10 mL solution with pH values of 3~9 and standing for 24 h before centrifugal drying. In addition, 20 mg Cu^2+^@Tb-MOFs powder was immersed in 10 mL aqueous solution and stood for 24 h and 48 h, respectively. Then the PXRD and luminescence spectra were measured.

The preparation of portable uric acid test paper: the filter paper (3 cm × 1 cm) was dipped in the dispersion of Cu^2+^@Tb-MOFs solution for 24 h, and then dried at room temperature.

## 4. Conclusions

In summary, a novel composite metal–organic framework Cu^2+^@Tb-MOFs is proposed for the detection of uric acid, a purine metabolite in living organisms. The prepared Cu^2+^@Tb-MOFs not only has good water stability and pH stability, but also has high sensitivity and anti-interference ability when other urine components coexist. The accurate determination of uric acid is achieved through the “turn on” fluorescence trigger mode, with a wide linear detection range (0~10^4^ µM) and a LOD as low as 0.65 µM, which improves the reliability of uric acid detection analysis and reduces the possibility of resulting false diagnosis. These results show that the composite material Cu^2+^@Tb-MOFs can be used as a promising fluorescence sensor for the detection of uric acid in urine. More importantly, a molecular logic gate was constructed, providing a promising technology for UA detection by intelligent control. This research may help to design other biochemical sensors and further open fluorescence applications in logic devices.

## Figures and Tables

**Table 1:** The performance of different probes to detect UA.

Probe	Work Range (μM)	LOD (μM)	Ref.
Eu-BDC@FM	0~200	0.6	[5]
MBP-SO_3_@Nafion	30~3000	11.3	[10]
CD-MONT-2	1.5~9.1	4.3	[14]
Hf-UiO-66-Py	0~30	1.4	[21]
Eu-TDA	0~327	0.689	[30]
MOF-235	10~90	3.46	[31]
Cu^2+^@MIL-91(Al:Eu)	0~1200	1.6	[45]
URICASE and HRP@HP-DUT-5	5~100	0.8	[46]
PCN-222(Fe)	10~800	3.5	[47]
Cu^2+^@Tb-MOFs	0~10^4^	0.65	This work

## Data Availability

Not applicable.

## Supplementary Materials

The following supporting information can be downloaded at: https://www.mdpi.com/article/10.3390/molecules27154803/s1, Characterization; Figure S1: (a) XRD patterns of Cu^2+^@Tb-MOFs in different pH aqueous solutions; (b) XRD patterns of Cu^2+^@Tb-MOFs after immersing in aqueous solutions for a few hours. Figure S2: (a) TGA of Tb-MOFs and Cu^2+^@Tb-MOFs samples; (b) DTG of Tb-MOFs and Cu^2+^@Tb-MOFs samples. Figure S3: (a) SEM and (b) EDX of Tb-MOFs samples. Figure S4: Emission (red line) spectra of as-prepared Cu^2+^@Tb-MOFs samples; emission (blue line) spectra of as-prepared Tb-MOFs samples. Figure S5: (a)The fluorescence intensity of Cu^2+^@Tb-MOFs at various immersion pH solutions; (b)The histogram of fluorescence intensity of Cu^2+^@Tb-MOFs at various immersion pH solutions; (c)The fluorescence intensity of Cu^2+^@Tb-MOFs at various immersion times; (d)The histogram of fluorescence intensity of Cu^2+^@Tb-MOFs at various immersion times. Figure S6: The fluorescence recovery response of UA to Cu^2+^@Tb-MOFs. Figure S7: The histogram of relative fluorescence intensity of Cu^2+^@Tb-MOFs at 545 nm after four recycles. Figure S8: The Chemical structural formula of uric acid. Figure S9: The fluorescence emission spectrum of Tb-MOFs (red), Cu^2+^@Tb-MOFs (blue), UA/Cu^2+^@Tb-MOFs (green) and UA/Tb-MOFs (purple), respectively. Figure S10: The corresponding photograph of samples under light (side view) and UV-light irradiation (top view), respectively: (a) Tb-MOFs; (b) Cu^2+^@Tb-MOFs; (c) UA/Cu^2+^@Tb-MOFs; (d) UA/Tb-MOFs. Figure S11: The UV-Vis absorption spectra of Cu^2+^ solution and mixed solution of Cu^2+^ and UA.

## Sample Availability

Samples of the compounds are available from the authors.
