# Supplementary material for: A Novel Turn-On Fluorescence Probe Based on Cu(II) Functionalized Metal–Organic Frameworks for Visual Detection of Uric Acid
Source: Molecules. 2022 Jul 27;27(15):4803. doi: 10.3390/molecules27154803 (PMC9369708; doi:10.3390/molecules27154803)
Supplement: Supplementary file 1 [file molecules-27-04803-s001.zip › molecules-1822856-supplementary.pdf]

Supplementary Materials

Characterization

Powder X-ray diffraction (XRD) measurements were performed on a Rigaku Smart Lab powder diffractometer employing Cu Kα radiation (λ=0.15406 nm). Fourier transform infrared spectroscopy (FTIR) spectrum was acquired on Nicolet NEXUS 670 spectrophotometer, using pressed KBr tables. The microstructures of all samples were examined on a FEI Quanta 250 scanning electron microscope. The fluorescence emission spectra were obtained on a Hitachi F-7000 fluorescence spectrophotometer equipped with a 150 W Xenon lamp as the excitation source. UV-vis adsorption spectra were recorded on a PE Lambda 950 spectrophotometer. X-ray photoelectron spectroscopy (XPS) was obtained by a ThermoFisher EscaLab 250Xi spectrophotometer. The contents of elements Tb and Cu were recorded by an PE OPTIMA8000 inductively coupled plasma optical emission spectrometry. Luminescence decay curves were measured by a Edinburgh Instruments F900 steady/transient state fluorescence spectrometer. All measurements were performed at room temperature.

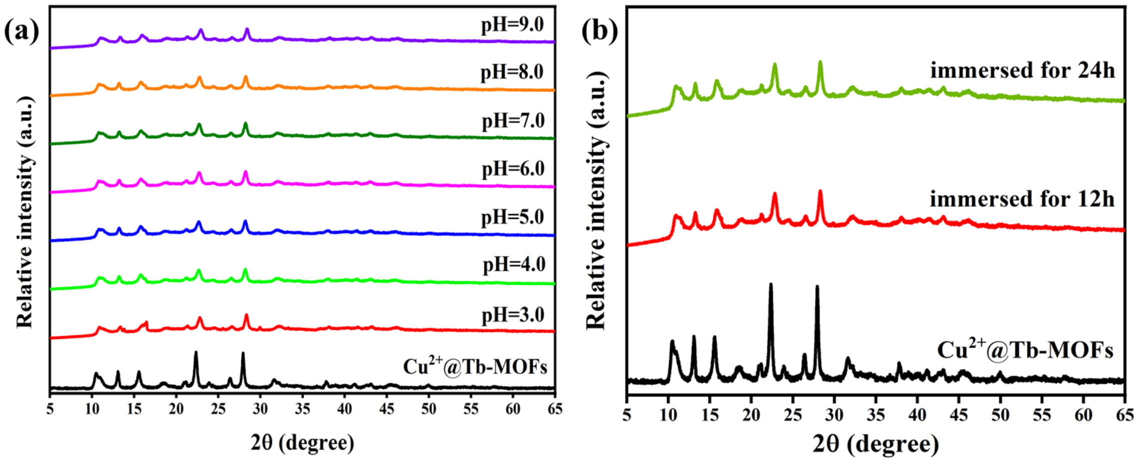

Figure S1. (a) XRD patterns of Cu^{2+}@Tb-MOFs in different pH aqueous solutions; (b) XRD patterns of Cu^{2+}@Tb-MOFs after immersing in aqueous solutions for a few hours.

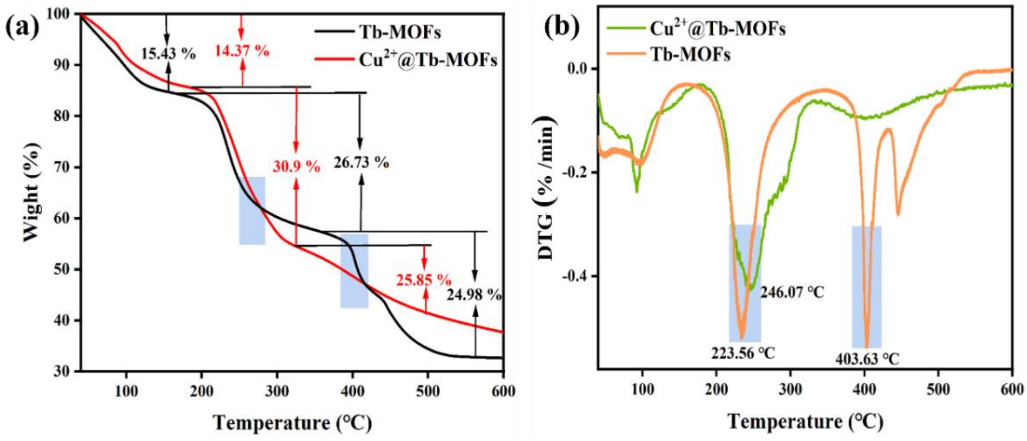

Figure S2. (a) TGA of Tb-MOFs and Cu^{2+}@Tb-MOFs samples; (b) DTG of Tb-MOFs and Cu^{2+}@Tb-MOFs samples.

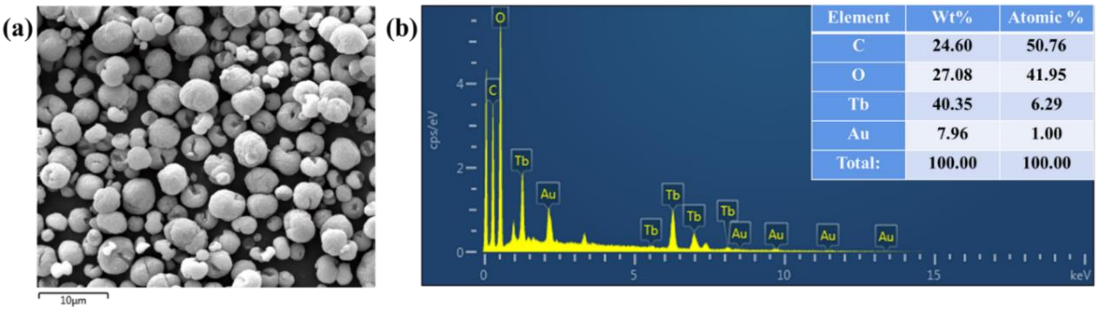

Figure S3. (a) SEM and (b) EDX of Tb-MOFs samples.

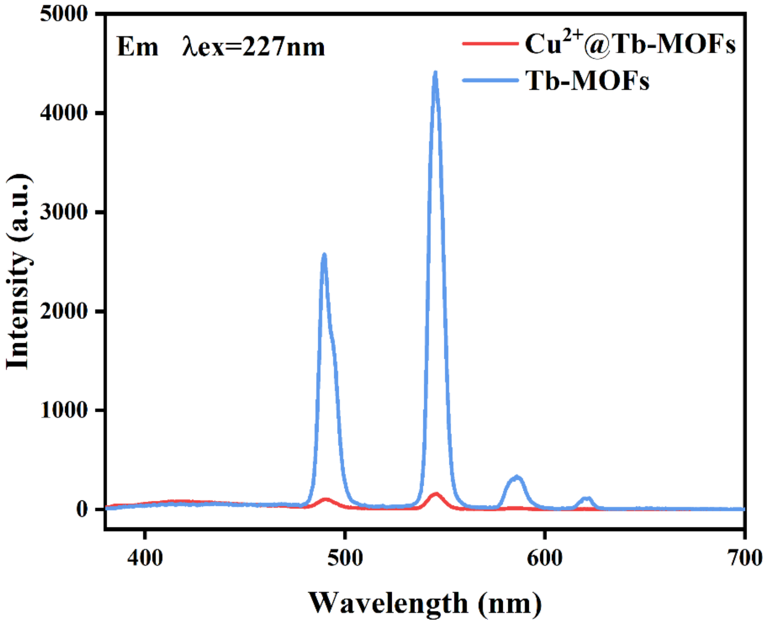

Figure S4. Emission (red line) spectra of as-prepared Cu^{2+}@Tb-MOFs samples; emission (blue line) spectra of as-prepared Tb-MOFs samples.

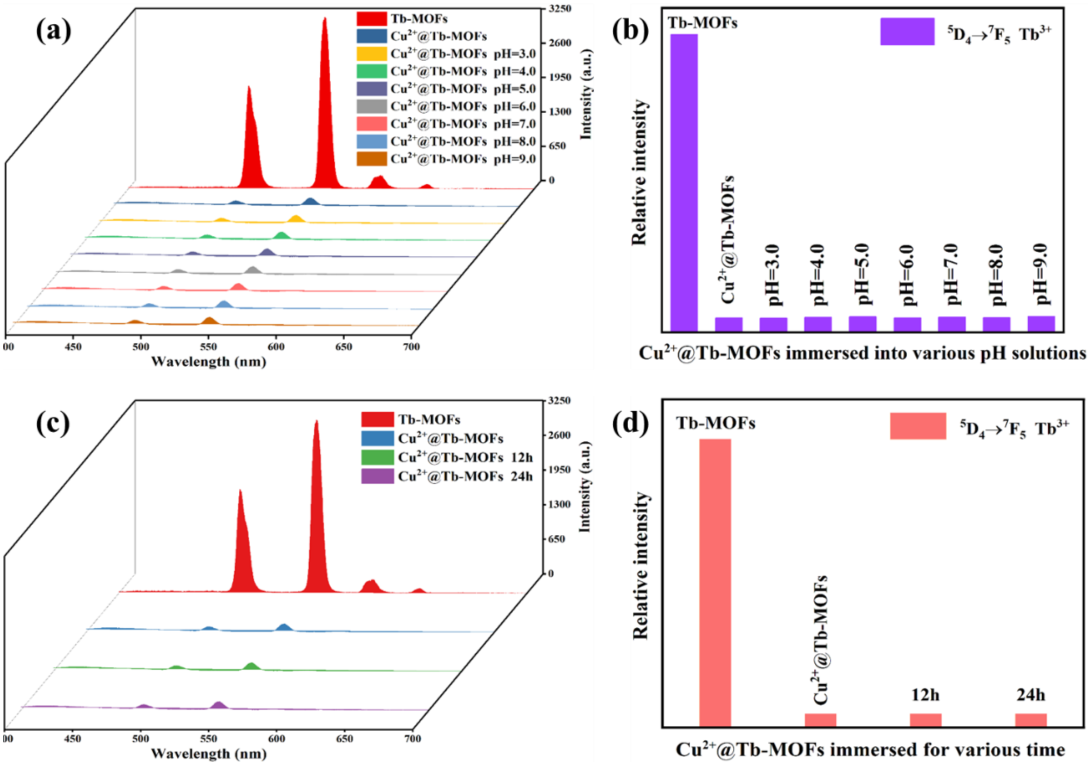

Figure S5. (a)The fluorescence intensity of Cu^{2+}@Tb-MOFs at various immersion pH solutions; (b)The histogram of fluorescence intensity of Cu^{2+}@Tb-MOFs at various immersion pH solutions; (c)The fluorescence intensity of Cu^{2+}@Tb-MOFs at various immersion times; (d)The histogram of fluorescence intensity of Cu^{2+}@Tb-MOFs at various immersion times.

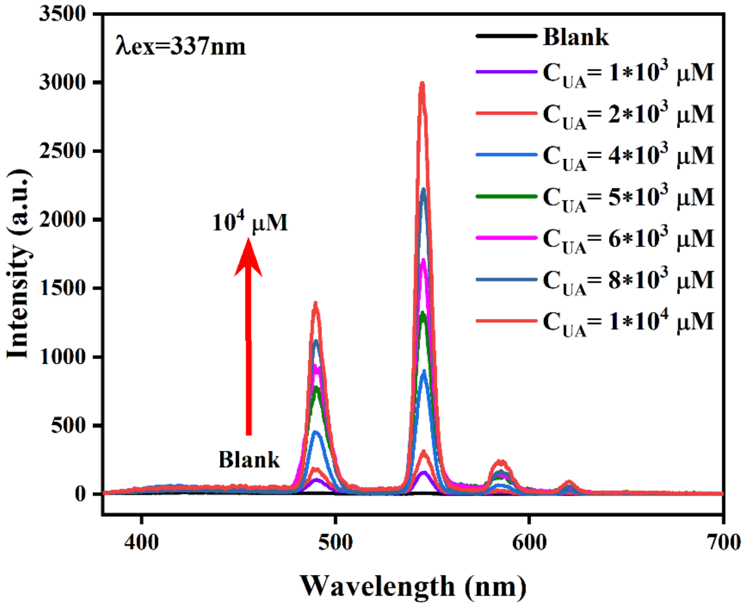

Figure S6. The fluorescence recovery response of UA to Cu^{2+}@Tb-MOFs.

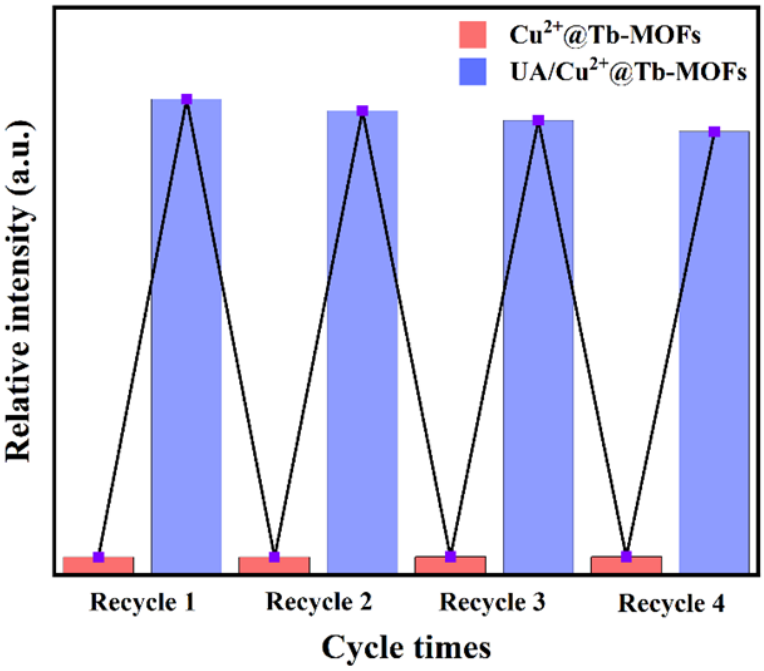

Figure S7. The histogram of relative fluorescence intensity of Cu^{2+}@Tb-MOFs at 545 nm after four recycles.

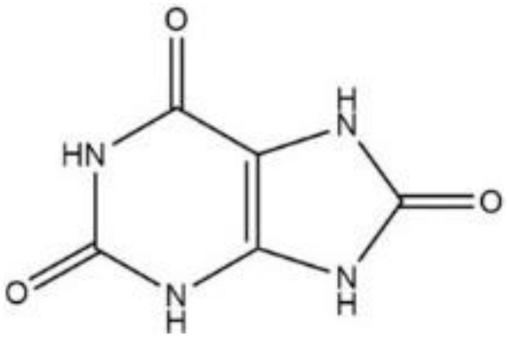

Figure S8. The Chemical structural formula of uric acid.

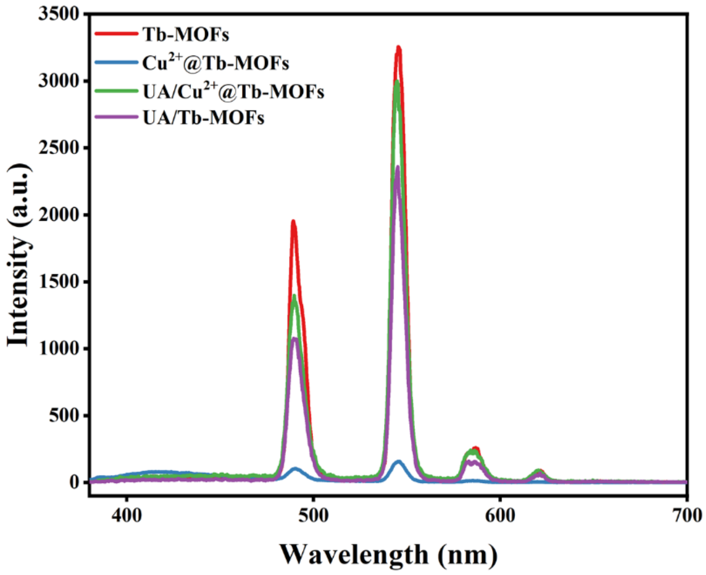

Figure S9. The fluorescence emission spectrum of Tb-MOFs (red), Cu^{2+}@Tb-MOFs (blue), UA/Cu^{2+}@Tb-MOFs (green) and UA/Tb-MOFs (purple), respectively.

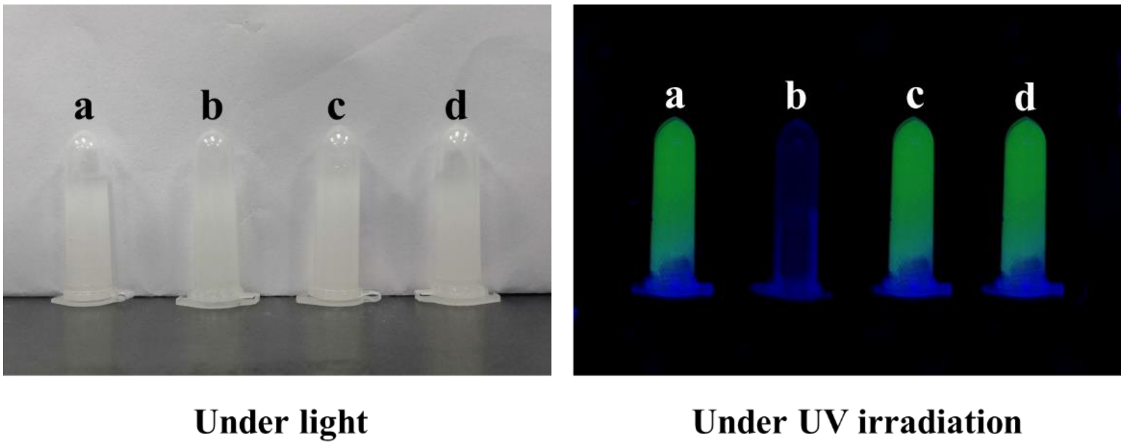

Figure S10. The corresponding photograph of samples under light (side view) and UV-light irradiation (top view), respectively: (a) Tb-MOFs; (b) Cu^{2+}@Tb-MOFs; (c) UA/Cu^{2+}@Tb-MOFs; (d) UA/Tb-MOFs.

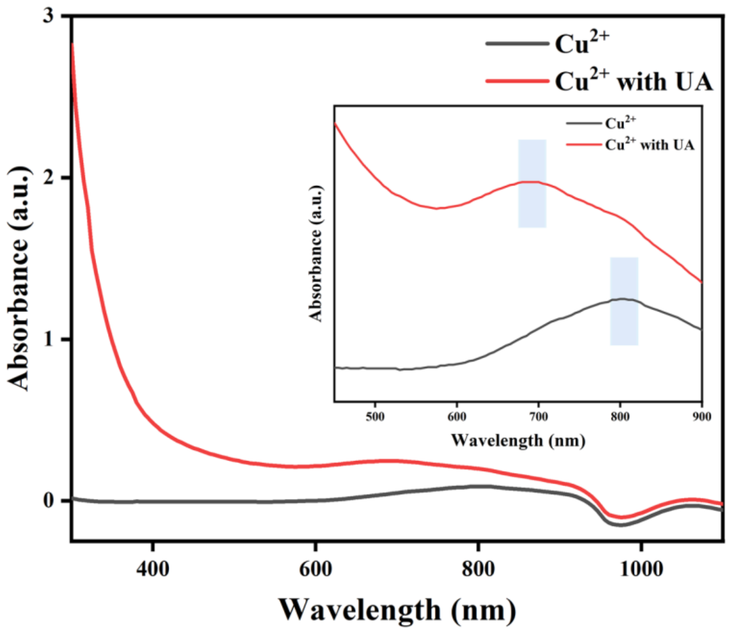

Figure S11. The UV-Vis absorption spectra of Cu^{2+} solution and mixed solution of Cu^{2+} and UA.
